# Complexity of arachnoid adhesions dictating the outcome of microvascular decompression for trigeminal neuralgia

**DOI:** 10.12669/pjms.40.12(PINS).11128

**Published:** 2024-12

**Authors:** Tariq Imran Khokar, Zulqarnain Akram Cheema, Ibreeza Fatima, Haseeb Mehmood Qadri, Raahim Bashir, Asif Bashir

**Affiliations:** 1 Dr. Tariq Imran Khokar, Associate Professor, Department of Neurosurgery, Unit-I, Punjab Institute of Neurosciences, Lahore, Pakistan; 2 Dr. Zulqarnain Akram Cheema, Post Graduate Resident, Department of Neurosurgery, Punjab Institute of Neurosciences, Lahore, Pakistan; 3 Dr. Ibreeza Fatima, Post Graduate Resident, Department of Neurosurgery, Punjab Institute of Neurosciences, Lahore, Pakistan; 4 Dr. Haseeb Mehmood Qadri, Post Graduate Resident, Department of Neurosurgery, Punjab Institute of Neurosciences, Lahore, Pakistan; 5 Dr. Raahim Bashir, Clinical and Research Intern, Department of Neurosurgery, Punjab Institute of Neurosciences, Lahore, Pakistan; 6 Prof. Dr. Asif Bashir, Professor, Department of Neurosurgery, Punjab Institute of Neurosciences, Lahore, Pakistan

**Keywords:** Trigeminal neuralgia, Pakistan, Arachnoiditis, Surgical adhesion

## Abstract

**Objective:**

Multiple techniques have been used to treat trigeminal neuralgia (TGN), including pharmacotherapy, radiosurgery, rhizotomy and microvascular decompression (MVD). Blood vessels are considered to be the most common cause of offense and compression to trigeminal nerve. We aimed to determine the causes of classic TGN and efficacy of MVD.

**Methods:**

This retrospective, cross-sectional study assessed the data of 53 patients, who underwent MVD at the Department of Neurosurgery, Punjab Institute of Neurosciences, Lahore, Pakistan, from May, 2022 to December, 2023. Information regarding patient demographics, clinical presentation, Barrow Neurological Institute Facial Pain Score (BNI–FPS), intra-operative findings, and postoperative complications were analyzed.

**Results:**

The mean age at presentation was 44.33 ± 11.71 years. Unilateral facial pain was the consistent clinical presentation among all patients, with right-sided involvement in 67.92% patients. All patients had involvement of at least two divisions of trigeminal nerve; maxillary and mandibular nerve in 84.91% and 71.70% cases, respectively. BNI–FPS Grade-IVb was found among 49.09% patients on presentation. Arachnoid adhesions, superior cerebellar artery and superior petrosal vein were the most common causes of compression in 71.70%, 50.94% and 43.40% cases, respectively. About 94% patients were discharged home on first post-operative day without complications and with BNI–FPS Grade-I.

**Conclusions:**

Microvascular decompression is highly advocated in classic cases of TGN. In the absence of grossly visible offending vascular structure, arachnoid adhesions should be the thorough focus of address.

## INTRODUCTION

Trigeminal neuralgia (TGN), also known as tic douloureux, is a facial pain disorder consisting of paroxysms of sharp, shooting, “lancinating” pain along the distribution of the trigeminal nerve, mostly involving its maxillary and mandibular divisions.^1^,^2^ Theories propose the pain to derive from focal demyelination of the trigeminal nerve secondary to compression from an aberrant artery or vein.^3^

Trigeminal neuralgia has an incidence of 4.7 persons per 100,000 with an average age of onset of 53 years and a sex predisposition towards women.^4^,^5^ Currently, there are three recognized types of this syndrome: classical, secondary, and idiopathic. Half of all patients suffer from the classical type; this is due to neurovascular contact resulting in structural changes in affected nerves.^5^ This contact has shown to be arterial in 82% of cases, with the remaining cases being venous or mixed arteriovenous.^6^ The most commonly culpable artery identified is the superior cerebellar artery, followed by the anterior inferior cerebellar artery. Conversely, the petrosal vein is the most frequent venous cause of neurovascular compression.^2^ Secondary trigeminal neuralgia is due to an underlying neurological disorder not resulting in neurovascular compression. This is most often a result of other demyelinating disorders, classically multiple sclerosis, but other conditions such as cerebellopontine angle tumor or arteriovenous malformations can also cause TGN. Finally, idiopathic trigeminal neuralgia is the diagnosis of exclusion when no other cause is found.^1^

Contemporary management of trigeminal neuralgia can be divided into medical, interventional, and surgical treatment options. First-line medical management consists of carbamazepine and oxcarbazepine, with gabapentin, pregabalin, and lamotrigine being acceptable second-line options.^5^ Balloon compression and glycerol injection are usually performed for idiopathic variety of TGN. The current neurosurgical gold standard is microvascular decompression (MVD).^5^ Newer interventions include stereotactic radiosurgery and radiofrequency rhizotomy.^7^

We perform MVD at our institution on patients with refractory classic trigeminal neuralgia and aim to elucidate the outcomes of MVD for treating classic TGN.

## METHODS

This retrospective, cross-sectional study was conducted at the Department of Neurosurgery, Punjab Institute of Neurosciences, Lahore, Pakistan in January 2024. We analyzed the records of 53 patients who underwent microvascular decompression for classic variety of trigeminal neuralgia pas an index surgery (primary cases) from May 01, 2022 to December 31, 2024, irrespective of their age and gender.

Information for data set of patient’s demographics, pre-operative Barrow Neurological Institute Facial pain Score (BNI–FPS), intra-operative findings and post-operative complications were collected and analyzed via Google Form (Google Inc., Mountainview, CA) and Microsoft Excel Sheet (Microsoft Corporation, Washington, United States), respectively, which was later sent to a statistician for descriptive analysis. Frequency and percentage were calculated for categorical data and mean with standard deviation was computed for continuous data.

### Ethical Approval Exemption:

The study was exempted from ethical approval by the Institutional Review Board of the mentioned hospital, reference # 1772/IRB/PINS/Approval/2024.

## RESULTS

The mean age of 53 patients was 44.33 ± 11.71 years, with male preponderance in 63.33% cases (Table-I). Facial pain was a complaint common to 100% patients, with an average symptom duration of 8.34 years. All patients in our study had at least two divisions of trigeminal nerve involved simultaneously (Table-II). Majority cases were right-sided. Many cases had more than one cause of compression of trigeminal nerve, with thick arachnoid adhesions found among 71.70% patients intra-operatively (Table-III and IV). BNI-FPS Grade-IV-b was most commonly noted among 49.09% of cases who opted for surgical intervention (Table-V). All patients were discharged home on first post-operative day without complications and with BNI–FPS Grade-I.

**Table-I:** Gender Distribution of Involved Patients with Trigeminal Neuralgia (TGN), where N= 53.

Gender	Number of Cases, n	Percentage Occurrence, n/N
Male	27	50.94%
Female	26	49.06%

**Table-II:** Number of Cases Involving Various Divisions of Trigeminal Nerve, where N= 53.

Divisions	Number of Cases, n	Percentage Occurrence, n/N
V_1_	21	39.62%
V_2_	45	84.91%
V_3_	38	71.70%

**Table-III:** Laterality of Cases with Trigeminal Neuralgia, where N= 53.

Laterality	Number of Cases, n	Percentage Occurrence, n/N
Right	36	67.92%
Left	17	32.08%

**Table-IV:** Causes of Compression of Trigeminal Nerve, where N= 53.

Cause of Compression	Number of Cases, n	Percentage Occurrence, n/N
Arachnoid Adhesions	40	71.70%
Superior Cerebellar Artery	27	50.94%
Superior Petrosal Vein	23	43.40%
Anterior Inferior Cerebellar Artery	10	18.87%
Duplicated Superior Cerebellar Artery	2	3.77%

**Table-V:** Pre-operative Barrow Neurological Institute Facial Pain Scale (BNI-FPS), where N= 53.

Grades	Number of Cases, n	Percentage Occurrence, n/N
I	-	-
II	4	7.54%
III a	7	13.20%
III b	7	13.20%
IV a	9	16.97%
IV b	26	49.09%

## DISCUSSION

The classic variety of TGN is empirically dealt via interventional procedures, including MVD, percutaneous destructive procedures, and stereotactic radiosurgery (SRS). Secondary cases associated with other pathologies like multiple sclerosis and cerebellopontine angle neoplasms are managed by percutaneous neurolysis or SRS, and surgery, respectively. SRS utilizes gamma knife (GK) or linear accelerator (LINAC) avoiding the risks of surgery and used in those who are medically unfit or unwilling to undergo a surgical procedure (C). A meta-analysis by Sharma et al. compares MVD with SRS, demonstrating provides a paramount success rate of 96% for MVD as compared to the success rate of 71.8% for GK-SRS.^8^ We recruited cases of classic TGN and hence made use of MVD at our center.

Facial pain is quantified with the Barrow Neurological Institute (BNI) grading scale: Grade-IV is severe pain refractory to medication, whereas Grade-I is regarded as an excellent clinical outcome featuring complete pain relief independent of medication use. About 66% of our patients had a pre-operative pain grading of BNI-FPS Grade-IV, similar to a Chinese study performed in 2020 had pre-op BNI-FPS Grade-IV in 55.98%.^2^ This clearly implies that patients opt for surgery when their symptoms are agonizingly unbearable and refractory to pharmacotherapeutic interventions. Several other pain grading scales, like visual analog scale (VAS), McGill Pain Questionnaire (MPQ) and Initiative on Methods, Measurement and Pain Assessment in Clinical Trials (IMMPACT) have been used other than BNI for trigeminal neuralgia. However, BNI-FPS allows for the measurement of both pain intensity and degree of medication use and is commonly used throughout the world.^9^,^10^

The mean patient age of our study was 44.33 ± 11.71 years, with a 50.94% male predominance. This is in contrast to a Spanish study by Pascasio et al. where 60 years was the average age, with 66% female patients. Shi et al. studied the risk factors for outcomes of MVD and documented a mean age of 59.86 ±10.81 years, with 63% males.^2^,^3^ Increasing aet rteriosclerosis and increasing atherosclerosis are bound to increasing age, leading to vessel tortuosity and deformation, causing trigeminal nerve compression. This reasoning is applicable to the sixth decade presentation of Spanish and Chinese studies, but for our patients, we think obesity is more closely related to atherosclerosis causing early presentation in the young ones than aging alone.^2^,^3^

All patients in our study had involvement of at least two of the three divisions of trigeminal nerve. The most commonly involved division was maxillary nerve, V2 in 84.91% of patients, followed by V3 and V1. Right-sided laterality was found in 69.20% patients in our study. The findings of a narrative review by British authors are consistent with our study, stating that 60% of their study patients had right-sided facial pain.^1^ This study also concluded that pain most commonly involves the V2 and V3 distribution with approximately 25% of patients having V1 involvement, corroborated by our results.^1^ Andersen et al. also showed that the most common involvement was mixed V2+V3 which is also consistent with our study.^5^

Since our research focused on MVD for classic trigeminal neuralgia secondary to neurovascular compression, we meticulously identified the offending vessels in all our patients undergoing surgery. The majority of our patients had more than one cause of trigeminal nerve compression. By far the most common etiology was arachnoid adhesions, with three-fourth of patients having evidence of this pathology. Adhesive arachnoiditis (AA) is usually due to viral, bacterial, or fungal infections, or secondary to subarachnoid hemorrhage, causing fibrosis and stiffening of the arachnoid mater followed by neural compression. This is usually in the spinal cord but can also affect the brain. However, it is interesting to note that all 40 of our patients with arachnoid adhesions had no brain or spinal cord pathology and normal blood/CSF counts.^11^ AA form an entangling complex surrounding pia matter. Pulsations transmitted via cerebrospinal fluid (CSF) and stretch changes in AA are indirectly transferred to trigeminal nerve leading to its hyperexcitability, and hence the symptoms of disease.^12^

The other causes of trigeminal nerve compassion in our patients were the superior cerebellar artery and superior petrosal vein. This is comparable to the previously discussed Chinese study in which 53.80%, 24.46%, and 5.98% of patients’ culpable vessels were the superior cerebellar artery, superior petrosal vein, and combined compression by the superior cerebellar and anterior inferior cerebellar arteries, respectively.^2^

Two of our patients had duplicated superior cerebellar artery as the offending vessel. According to a 2019 Japanese case report, this congenital condition has an average unilateral incidence rate of 19.5%, but is the cause of offense in only 0.8% of trigeminal neuralgia cases.^13^

Out of the 53 patients we enrolled in this study, four had undergone prior rhizotomy with poor post-procedural outcome for their facial pain. After MVD, their pain severity was at BNI-FPS Grade-I along with the other 49 patients, implying the superiority of MVD over SRS. Partial sensory rhizotomy (PSR) is done in cases of secondary or idiopathic trigeminal neuralgia when there is no neurovascular compression, and it may be performed along with MVD in cases of compression.^7^

None of our patients had any postoperative complications such as wound dehiscence, stroke, hemorrhage, hearing loss, hypesthesia or facial weakness, all of which are routinely encountered complications of trigeminal neuralgia surgery.^7^ Although CSF leak and wound infections were noted in 1.89% and 3.78% patients, respectively. Another local study conducted at Lady Reading Hospital, Peshawar demonstrates a CSF leak and wound infection rate of 8.34% and 5.40%, respectively.^14^

Postoperatively, 100% of our patients had complete pain relief on their first post-op day consistent with BNI-FPS Grade-I. This is in comparison with the previously mentioned Chinese study where 72.28% of patients had a post-op pain Grade of I-II, but 27.72% had inadequate or recurring facial pain of grades >II.^2^ This is also similar to another study performed in Spain where 97% of cases had significant pain relief on the first post-op day with BNI-FPS Grades I-III.^3^ A previous study by Rehman et al. in 2012 also reveals a 92% immediate relief of trigeminal neuralgia following MVD at our center.^15^ Muazzucchi et al. concluded the poor prognosis of trigeminal neuralgia in patients with focal arachnoiditis, finding focal arachnoiditis in 13% patients of their studied cases.^16^

According to our study, arachnoid adhesions are the most common culprit in trigeminal nerve compression and they should be dissected completely. It is not necessary to find culpable vessels in every case. Meticulous surgical technique and knowledge of offending causes is fundamental in achieving substantially fruitful results.

### Limitations:

The retrospective nature of the study, small sample size and mono-centricity of the study are apparent limitations of the study. Arachnoid adhesions were not sent for histopathological or microbiological analysis to find out the cause of their development. Authors have planned another study to address the cause of arachnoid adhesions.

## CONCLUSION

Microvascular decompression is highly advocated in classic cases of TGN. Precise placement of synthetic polymers between the offending vessel and TGN and aseptic technique produce remarkable clinical outcomes, as in our study. Arachnoid adhesions were the most common cause of TGN compression at our center, with maxillary involvement in majority cases. We advocate the identification of arachnoid adhesions and their complete lysis in cases where they ae found. The utilization of BNI–FPS as a marker of preoperative and postoperative severity assessment is effective.

### Authors Contribution:

**TIK** conceived the study, wrote the manuscript draft, critically reviewed the article and is responsible and accountable for the accuracy or integrity of the work.

**ZAC and IF** contributed to literature search, data collection and manuscript writing.

**HMQ** designed the study, did literature search, wrote the manuscript draft and critically reviewed the article.

**RB** contributed to literature review and manuscript writing.

**AB** supervised the project and critically reviewed the article.

All authors approve the final version of the manuscript.
